# Supplementary material for: The GENDULF algorithm: mining transcriptomics to uncover modifier genes for monogenic diseases
Source: Mol Syst Biol. 2020 Dec 8;16(12):e9701. doi: 10.15252/msb.20209701 (PMC7754056; doi:10.15252/msb.20209701)
Supplement: Supplementary file 1 — Appendix [file MSB-16-e9701-s001.docx]

**Table of Contents**

| **Appendix Tables** | |
| --- | --- |
| **Table S1** | Case-control sample count for CF and SMA |
| **Table S2** | Human muscle and spinal cord case information |
| **Table S3** | siRNA/SSO Sequence or Catalog Number |
| **Table S4** | Custom Taqman Probes for qPCR detection |
| **Table S5** | Commercially available Taqman Assays used for qPCR detection |
| **Table S6** | SYBR Green primers used as reference controls in qBase+ GeNorm analysis |
| **Table S7** | Antibodies and dilutions used for western blot |

| **Appendix Figures** | |
| --- | --- |
| **Figure S1** | Sensitivity and specificity analysis for CF lung tissue modifiers |
| **Figure S2** | Power analysis for CF lung tissue modifiers |
| **Figure S3** | GENDULF step 1 evaluation for loci found associated with CF phenotype |
| **Figure S4** | Evidence of compensating SMN2 exon 7 inclusion for low levels of SMN1 in healthy tissues |
| **Figure S5** | GENDULF candidate controls for SMA |
| **Figure S6** | GENDULF step 2 p-values for CF lung tissue |

**Appendix Expanded View Discussion**

**Appendix Figure S1**

**Figure S1. Sensitivity, specificity and positive predictive value (PPV) analysis for CF lung tissue modifiers.** Bar plots showing the sensitivity **(A)**, specificity **(B)** and positive predictive value (PPV) **(C)** for GENDULF predicted CF modifiers in lung tissues, obtained with different thresholds for step1 (X-axes), when applied to protein coding genes. In this calculation of PPV, the true positives are predicted modifiers reported as modifiers in the literature, while the false positives are predicted modifiers not reported in the literature. This form of PPV is expected to increase over time as more modifiers are validated, as exemplified by the recent validations of *KRT8* and *MUC1* (Shanthikumar et al. 2019) Blue bars are for GENDULF step1, and green for step 2. The dashed line in panels A and C represents the random sensitivity and PPV. The sensitivities for both steps of GENDULF with the 0.1 threshold are increases of over 200-fold compared to the random rate. The PPV increases in over 2-fold from GENDULF step1 to step 2 for every considered threshold for step1. The P-values in panel (A) are hypergeometric enrichment P-values.

**Appendix Figure S2**

**Figure S2. Power analysis for CF lung tissue modifiers.** **(A)** boxplots of the distribution of power over CF lung modifiers predicted in GENDULF step 1, with different number of case and control samples (x-axes). **(B)** bar plots showing the average with standard deviation error bars of the confidence levels obtained for the PMs of Lung tissue with CFTR as GCD, for different numbers of positive and negative samples (X-axis).

**Appendix Figure S3**

**
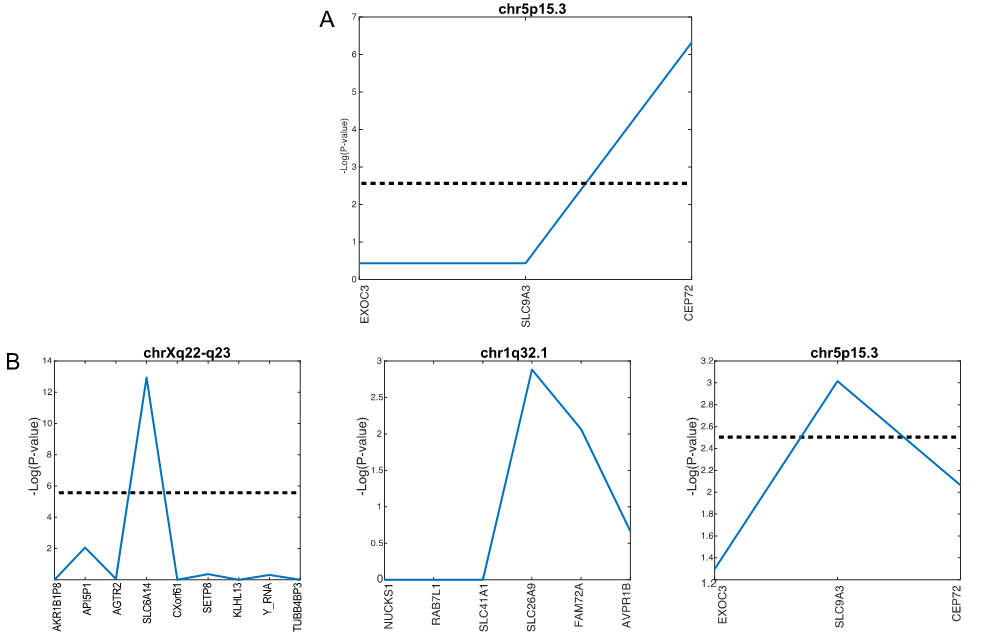
**

**Figure S3.** GENDULF step 1 evaluation for loci found associated with CF phenotype. (A) The p-values assigned by GENDULF step 1, applied to healthy GTEx lung tissues, to genes within chr5p15.3 chromosomal segment, ordered by their location. The dashed line represents a significance threshold corrected for the number of genes evaluated (B) The p-values assigned by GENDULF step 1, applied to healthy GTEx colon tissues, to genes within chrXq22-q23, chr6p21.3 and chr5p15.3 chromosomal segments, ordered by their location. The dashed line represents a significance threshold corrected for the number of genes evaluated.

**Appendix Figure S4**

**Figure S4. Evidence of compensating SMN2 exon 7 inclusion for low levels of SMN1 in healthy tissues. (A)** Scatter plot showing SMN1 levels (x-axis) vs. the ratio between SMN2-FL to SMN2Δ7 in healthy muscle tissue. **(B)** Scatter plot showing SMN1 levels (x-axis) vs. the ratio between SMN2-FL to SMN2Δ7 in healthy spinal cord tissues. **(C)** The levels of HNRNPA1 HNRNPC and KHDRBS1 modifiers that enhance exon 7 inclusion when downregulated in muscle samples with low ratio of SMN2-FL to SMN2∆7 and those with high ratio of SMN2-FL to SMN2∆7 (Pedrotti *et al*, 2010; Irimura *et al*, 2009).

**Appendix Figure S5**

**Figure S5. GENDULF candidate controls for SMA. (A)-(B)** Scatter plots associating the expression of the GCD (SMN1) vs. identified PMs in healthy GTEx muscle tissues (left panels) and spinal cord tissues (right panels); the expression of SMN1 (X-axis) vs. that of SF3B3 (A) and NECAP1 (B). Bottom Panels: Boxplots associating the expression of the identified DPMs in case-control studies; **(C)-(D)** the expression of SF3B3 (C) and NECAP1 (D) in SMA and in healthy control muscle (left panels) and spinal cord (middle panels) tissues and the levels of these predicted modifiers healthy muscle samples with low ratio of SMN2-FL to SMN2∆7 and those with high ratio of SMN2-FL to SMN2∆7 (right panels).

**Appendix Figure S6**

**
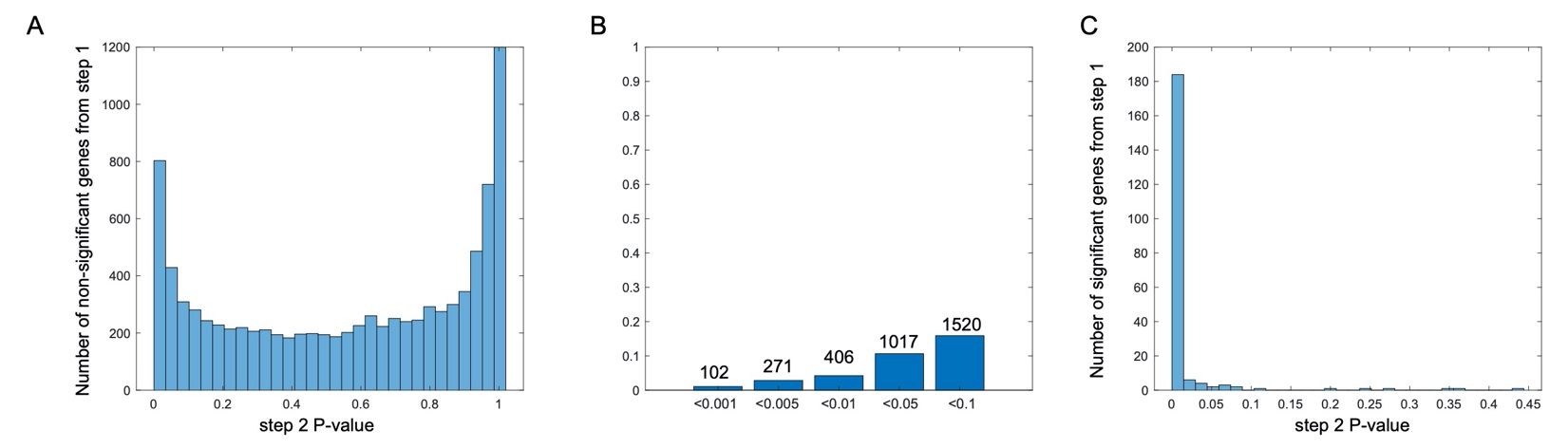
**

**Figure S6. GENDULF step 2 p-values for CF and lung tissue. (A)** histogram showing the null distribution of GENDULF step 2 p-values assigned to genes that were not found significant via GENDULF step1. **(B)** Bar plot showing the percentage of significant GENDULF step 2 p-values assigned to genes that were not found significant via GENDULF step1 (y-axis), for different thresholds of p-values (x-axis). The numbers above the bars are the number of genes passing each p-value threshold (out of a total of 9560 with GENDULF step1 p-value >0.05) **(C)** histogram showing the distribution of GENDULF step 2 p-values assigned to genes that were found significant via GENDULF step 1.

**Appendix Table S1 Case-control sample count for CF and SMA**

| Disease | Tissue | Number of Case (disease) samples | Number of control samples |
| --- | --- | --- | --- |
| CF | Lung | 9(4 mild) | 11 |
| CF | Intestine | 16 | 13 |
| SMA | Muscle | 11 | 18 |
| SMA | Spinal cord | 6 | 12 |

**Appendix Table S2. Human muscle and spinal cord case information**

| Case ID | SMA/  Control | Age  (months) | SMN1  Copies | SMN2  Copies | PMI (hours) | Cause of death | Spinal  cord | Iliopsoas  muscle | Diaphragm  muscle |
| --- | --- | --- | --- | --- | --- | --- | --- | --- | --- |
| SMA_11_01 | SMA | 1.8 | 0 | 2 | 7 | Type I SMA | X |  |  |
| SMA_08_02 | SMA | 16 | 0 | 2 | 7 | Type I SMA | X | X | X |
| SMA_12_01 | SMA | 2.5 | 0 | 2 | 7 | Type I SMA |  | X | X |
| SMA_08_01 | SMA | 4.5 | 0 | 2 | 14.5 | Type I SMA | X | X | X |
| SMA_09_02 | SMA | 4 | 0 | 2 | 4 | Type I SMA | X | X | X |
| SMA_10_16 | SMA | 72 | 0 | 2 | 24 | Type II SMA | X |  | X |
| SMA_14_04 | SMA | 72 | 0 | 2 | 24 | Type I SMA | X | X | X |
| CTL_12_02 | Control | 0.3 | 2 | 2 | 26 | Meconium Aspiration |  | X | X |
| CTL_95_04 | Control | 0.3 | 2 | 2 | 8 | Congenital heart disease | X |  |  |
| CTL_90_08 | Control | 1.3 | 2 | 2 | 8 | Arthrogryposis | X |  |  |
| NBB_25 | Control | 1.6 | 2 | 1 | 24 | SIDS | X |  |  |
| NBB_18 | Control | 1.9 | 2 | 1 | 20 | SIDS | X |  |  |
| NBB_86 | Control | 1.9m | 2 | 2 | 11 | Congenital Heart Defect |  |  | X |
| NBB_657 | Control | 2 | 2 | 2 | 15 | SIDS | X |  |  |
| NBB_1296 | Control | 3.26 | 2 | 2 | 16 | Control |  | X |  |
| NBB_1472 | Control | 3.93m | 2 | 2 | 19 | Control |  |  | X |
| NBB_195 | Control | 4.1 | 2 | 2 | 16 | Control |  | X |  |
| CTL_93_07 | Control | 14 | 2 | 1 | 8 | Liver disease-coagulopathy | X |  |  |
| CTL_12_05 | Control | 19 | 2 | 1 | 19 | Unknown |  | X | X |
| CTL_13_01 | Control | 168 | 2 | 1 | 2 | Cardiac Arrest | X | X | X |

**Appendix Table S3. siRNA/SSO Sequence or Catalog Number**

| siRNA/ASO Name | Sequence or Catalog Number |
| --- | --- |
| SMN2 SSO | 5’‐ATTCACTTTCATAATGCTGG‐3’ |
| U2AF1 | ON-TARGETplus Human U2AF1 siRNA (Dharmacon, L-012325-01-0005) |
| HNRNPA0 | ON-TARGETplus Human HNRNPA0 siRNA (Dharmacon, L-012314-02-0005) |
| SF3B3 | ON-TARGETplus Human SF3B3 siRNA (Dharmacon, L-020085-00-0005) |
| NECAP1 | ON-TARGETplus Human NECAP1 siRNA (Dharmacon, L-017872-01-0005) |
| Scrambled siRNA (Non-targeting) | ON-TARGETplus Non-targeting Control Pool (Dharmacon, D-001810-10-05 5 nmol) |

**Appendix Table S4. Custom Taqman Probes for qPCR detection**

| Name | Forward Primer | Reverse Primer | Probe (Taqman) |
| --- | --- | --- | --- |
| Total SMN (SMN12) | CAG GAG GAT TCC GTG CTG TT | CGG CAC AGG CCA GAG CGA TG | TCA GTG CTG TAT CAT CCC AAA TG |
| SMN1-FL | TAT CAT ACT GGC TAT TAT ATG GGT TTC | AAG GAG AAA TGC TGG CAT AGA GCA GC | TCG TTT CTT TAG TGG TGT CAT TTA G |
| SMN2-FL | TAT CAT ACT GGC TAT TAT ATG GGT TTT | AAG GAG AAA TGC TGG CAT AGA GCA GC | TCG TTT CTT TAG TGG TGT CAT TTA G |
| SMN2∆7 | TGG CTA TCA TAC TGG CTA TTA TAT GGA A | CTG GCA TAG AGC AGC ACT AAA TGA CAC CAC | TCC AGA TCT GTC TGA TCG TTT CTT |

**Appendix Table S5. Commercially available Taqman Assays used for qPCR detection**

| Name | Catalog Number |
| --- | --- |
| U2AF1 | Hs00739599_m1 (Thermo Fisher) |
| HNRNPA0 | Hs00246543_s1 (Thermo Fisher) |
| SF3B3 | Hs00418633_m1 (Thermo Fisher) |
| NECAP1 | Hs00747865_m1 (Thermo Fisher) |

**Appendix Table S6. SYBR Green primers used as reference controls in qBase+ GeNorm analysis**

| Name | Forward Primer | Reverse Primer |
| --- | --- | --- |
| ACTB | GAC GAC ATG GAG AAA ATC TG | ATG ATC TGG GTC ATC TTC TC |
| ATP5B | TAC CAC CAA TTC TAA ATG CC | GTG CTC TCA CCC AAA TG |
| B2M | AAG GAC TGG TCT TTC TAT CTC | GAT CCC ACT TAA CTA TCT TGG |
| EIF4A2 | AGA GAG ATG TTA TCA TGA GGG | TAA CCA AAG ACA CTT GTT GC |
| FBXO38 | AAA GAA GAT GCC AGA TGT TG | AAA TGA GAA GTT TCC ACA CC |
| GAPDH | ACA GTT GCC ATG TAG ACC | TTT TTG GTT GAG CAC AGG |
| UBC | CGT CAC TTG ACA ATG CAG | TGT TTC CAG CAA AGA TCA G |
| YWHAZ | AACT TGA CAT TGT GGA CAT C | AAA ACT ATT TGT GGG ACA CGC |

**Appendix Table S7. Antibodies and dilutions used for western blot**

| Antibody Name | Company (Catalog Number) | Dilution |
| --- | --- | --- |
| Mouse anti-SMN | BD Transduction Laboratories (610646) | 1:1,000 |
| Rabbit anti-U2AF1 | Cell Signaling Technology (13705) | 1:1,000 |
| Mouse anti-GAPDH | ThermoFisher (AM4300) | 1:5,000 |
| Goat anti-rabbit IgG – AP | Sigma (A3687-.5ml) | 1:5,000 |
| Goat anti-mouse IgG – AP | Sigma (A3562-1ML) | 1:5,000 |

**Appendix Expanded View Discussion**

We explored the literature to find diseases in which mutations or reduced expression of a second gene are associated with a more severe phenotype, in addition to CF and SMA. Variants leading to reduced expression of *BCL11A* mitigating the severity of sickle-cell anemia (caused by mutations in *HBB*) are the best-known non-CF, non-SMA examples of a gene modifier for a recessive disease in which reduced expression of the modifier mitigates disease severity and hence, fits exactly the GENDULF assumptions (Uda *et al*, 2008; Sankaran *et al*, 2008). (Deficiency of) *KLF1* is another modifier of sickle-cell anemia acting in the same direction (Borg *et al*, 2010). The related disorder β-thalassemia is less severe in patients who have a deletion at the unlinked HBA locus (Higgs et al., 1982; Embury et al., 1982). The main molecular effect of the *BCL11A* and *KLF1* modifiers is to increase the expression of fetal hemoglobin showing that the disease-specific, transcript-specific Step 3 in GENDULF is relevant for diseases other than SMA.

Other recessive diseases in which reduced expression of a modifier is associated with less severe disease include: *CCND1* as a modifier for von Hippel-Lindau disease (Zatyka *et al*, 2002), *MMP1* for dystrophic epidermolysis bullosa (Zatyka *et al*, 2002), and *ACE* for glycogen storage disease type V (McArdle disease (Martinuzzi *et al*, 2003; Suehiro *et al*, 2004)). GENDULF might be applied to these diseases, as well as to autosomal dominant diseases in which reduced expression of modifiers is associated with less severe disease, such as *CNOT1* as a modifier of the retinitis pigmentosa caused by mutations in *PRF31* (Venturini *et al*, 2012), *SMAD6* for craniosynostosis (Timberlake *et al*, 2016), and *FIGN* for pulmonary arterial hypertension (Puigdevall *et al*, 2019).

**Appendix References**

Borg J, Papadopoulos P, Georgitsi M, Gutiérrez L, Grech G, Fanis P, Phylactides M, Verkerk AJMH, van der Spek PJ, Scerri CA et al (2010) Haploinsufficiency for the erythroid transcription factor KLF1 causes hereditary persistence of fetal hemoglobin. *Nat. Genet.* 42: 801-805

Embury SH, Dozy AM, Miller J, Davis JR, Kleman KM, Preisler H, Vichinsky E, Lande WN, Lubin BH, Kan YW et al (1982) Concurrent sickle-cell anemia and α-thalassemia: Effect on severity of anemia. *N. Engl. J. Med.* 306: 270-274

Higgs DR, Aldridge, BE, Lamb, J., Clegg, J.B., Weatherall, D.J., Hayes, R.J., Grandison Y, Lowrie, Y., Mason, K.P., Serjeant, B.E et al (1982) Interaction of alpha-thalassemia and homozygous sickle-cell disease. *N. Engl. J. Med.* **306***:* 1441-1446

Irimura S, Kitamura K, Kato N, Saiki K, Takeuchi A, Gunadi, Matsuo M, Nishio H & Lee MJ (2009) HnRNP C1/C2 may regulate exon 7 splicing in the spinal muscular atrophy Gene SMN1. *Kobe J. Med. Sci.* 54: E227-E236

Martinuzzi A, Sartori E, Fanin M, Nascimbeni A, Valente L, Angelini C, Siciliano G, Mongini T, Tonin P, Tomelleri G, et al (2003) Phenotype modulators in myophosphorylase deficiency. *Ann. Neurol.* 53:497-502

Pedrotti S, Bielli P, Paronetto MP, Ciccosanti F, Fimia GM, Stamm S, Manley JL, Sette C (2010) The splicing regulator Sam68 binds to a novel exonic splicing silencer and functions in SMN2 alternative splicing in spinal muscular atrophy. *EMBO J.* 29: 1235–1247

Puigdevall P, Piccari L, Blanco I, Barberà JA, Geiger D, Badenas C, Milà M, Castelo R, Madrigal I (2019) Genetic linkage analysis of a large family identifies FIGN as a candidate modulator of reduced penetrance in heritable pulmonary arterial hypertension. *J. Med. Genet.* 56:481-490

Sankaran VG, Menne TF, Xu J, Akie TE, Lettre G, Van Handel B, Mikkola HKA, Hirschhorn JN, Cantor AB, Orkin SH (2008) Human fetal hemoglobin expression is regulated by the developmental stage-specific repressor BCL11A. *Science* 322:1839-1842

Shanthikumar S, Neeland MN, Saffery R, Ranganathan S (2019) Gene modifiers of cystic fibrosis lung disease: A systematic review. Pediatr. Pulmon. 54: 1356-1366

Suehiro T, Morita T, Inoue M, Kumon Y, Ikeda Y. Hashimoto K (2004) Increased amount of the angiotensin-converting enzyme (ACE) mRNA originating from the ACE allele with deletion. *Hum. Genet.* **115**:91-96

Timberlake AT, Choi J, Zaidi S, Lu Q, Nelson-Williams C, Brooks ED, Bilguvar K, Tikhonova I, Mane S, Yang JF, et al (2016) Two locus inheritance of non-syndromic midline craniosynostosis via rare *SMAD6* and common *BMP2* alleles. *Elife* **5**:pii:20125

Uda M, Galanello R, Sanna S, Lettre G, Sankaran VG, Chen W, Usala G, Busonero F, Maschio A, Albai G et al (2008) Genome-wide association study shows *BCL11A* associated with persistent fetal hemoglobin and amelioration of the phenotype of β-thalassemia. *Proc. Natl. Acad. Sci. U. S. A.* **105**:1620-1625

Venturini G, Rose AM, Shah AZ, Bhattacharya SS & Rivolta C (2012) *CNOT3* Is a modifier of *PRPF31* mutations in retinitis pigmentosa with incomplete penetrance. *PLoS Genet.* **8**:e1003040

Zatyka M, Da Silva NF, Clifford SC, Morris MR, Wiesener MS, Eckardt KU, Houlston RS, Richards FM, Latif F, Maher ER (2002) Identification of cyclin D1 and other novel targets for the von Hippel-Lindau tumor suppressor gene by expression array analysis and investigation of cyclin D1 genotype as a modifier in von Hippel-Lindau disease. *Cancer Res.* **62**:3803-3811
